# Building a competent health manager at district level: a grounded theory study from Eastern Uganda

**DOI:** 10.1186/s12913-016-1918-0

**Published:** 2016-11-21

**Authors:** Moses Tetui, Anna-Karin Hurtig, Elizabeth Ekirpa-Kiracho, Suzanne N. Kiwanuka, Anna-Britt Coe

**Affiliations:** 1 Makerere University School of Public Health (MakSPH), Makerere University, New Mulago Complex, P.O. B0X 7072, Kampala, Uganda; 2 Epidemiology and Global Health Unit, Department of Public Health and Clinical Medicine, Umeå University, Umeå, SE 90185 Sweden

**Keywords:** Health management, Capacity building, Health systems, Low-income settings, District-level, Uganda, Grounded theory method

## Abstract

**Background:**

Health systems in low-income countries are often characterized by poor health outcomes. While many reasons have been advanced to explain the persistently poor outcomes, management of the system has been found to play a key role. According to a WHO framework, the management of health systems is central to its ability to deliver needed health services. In this study, we examined how district managers in a rural setting in Uganda perceived existing approaches to strengthening management so as to provide a pragmatic and synergistic model for improving management capacity building.

**Methods:**

Twenty-two interviews were conducted with district level administrative and political managers, district level health managers and health facility managers to understand their perceptions and definitions of management and capacity building. Kathy Charmaz’s constructive approach to grounded theory informed the data analysis process.

**Results:**

An interative, dynamic and complex model with three sub-process of building a competent health manager was developed. A competent manager was understood as one who knew his/her roles, was well informed and was empowered to execute management functions. Professionalizing health managers which was viewed as the foundation, the use of engaging learning approaches as the inside contents and having a supportive work environment the frame of the model were the sub-processes involved in the model. The sub-processes were interconnected although the respondents agreed that having a supportive work environment was more time and effort intensive relative to the other two sub-processes.

**Conclusions:**

The model developed in our study makes four central contributions to enhance the WHO framework and the existing literature. First, it emphasizes management capacity building as an iterative, dynamic and complex process rather than a set of characteristics of competent managers. Second, our model suggests the need for professionalization of health managers at different levels of the health system. Third, our model underscores the benefits that could be accrued from the use of engaging learning approaches through prolonged and sustained processes that act in synergy. Lastly, our model postulates that different resource investments and a varied range of stakeholders could be required at each of the sub-processes.

## Background

Health systems in low-income countries have long been characterized by poor health outcomes [1–3]. While much effort has been made to improve health systems, many challenges remain, among these is weak management capacity [4, 5]. According to the World Health Organization (WHO), the management of health systems interventions is as critical as their implementation in order to sustain improvements of public health outcomes [6, 7]. Management is a social discipline concerned with the behavior of people within social institutions as influenced by policies, structures, processes, values and the context within which it is practiced [8, 9]. According to Rockers and Barnighausen, management is the glue that ensures the proper functioning of different components or building blocks of the health system [10]. The need to have competent managers at all levels of the health systems can therefore not be overstated.

In its efforts to emphasize the crucial role that management plays in enabling health systems, the WHO developed a framework comprised of four key conditions needed to ensure strong management of health systems. These included having: an adequate number of trained managers, managers with appropriate competencies, management support systems and an enabling work environment [7].

First, according to the WHO framework, efforts should be undertaken to guarantee the existence of sufficient numbers of qualified health managers and their equitable distribution at all levels of the health system in a country [7]. In low-income countries, health managers in the public sector are often medical, clinical or nursing personnel assigned to this as an extra role. They have to balance between clinical and managerial work which is often complex and difficult [11]. In terms of numbers and distribution, duly qualified health managers are in short supply and concentrated in urban settings and in the private sector [12]. Moreover, capacity building of health managers in low-income countries receives relatively little attention. Management structures and competencies at the district or sub national level are even weaker [10, 13]. Meanwhile, district level managers are significant for the functioning of the health system, especially in settings that have undergone decentralization [14, 15]. To ensure adequate numbers and distribution of health managers, several strategies have been employed, ranging from policy legalization to training programs with varying degrees of implementation and success [16, 17].

In the WHO framework, the second condition for strong management of health systems is appropriateness of management competencies [7]. Management competencies are typically described in relation to the term “capacity”, which refers to individual attributes that enable or hinder managers’ performance [18, 19]. According to the literature, managers at the district level are more likely than those at the national level to lack the necessary skills, attitudes and behaviors needed to perform management duties [11, 20]. Several approaches have been advanced and implemented to strengthen managers’ competencies including formal training, on-the job training, action learning and non-formal training [21]. These approaches offer health managers valuable opportunities for learning, practice and reflection, which in turn increase their knowledge and improves their skills [10, 22]. Nonetheless, these approaches are often implemented in insolation from one another rather than in combination [17]. As shown in existing literature, no one approach is sufficient to build management capacity. Building management capacity has been shown to be more, iterative, dynamic and complex, which cannot be achieved by any one approach [4, 23].

Thirdly, the WHO framework recommends core management support systems as a key condition for ensuring strong management for health systems [7]. These support systems are comprised of planning and budgeting, financial management, supplies and logistics, infrastructure management, personnel management and health information and monitoring systems. Moreover, such support systems require competent personnel to run them [7]. In low-income countries, while these systems are readily available, their capacity is often low, at sub national levels these support systems are weakest [6, 11, 24].

Lastly, health managers need an enabling environment to competently perform their roles [7]. Regular meetings, support supervision and mentoring, incentives, organizational support structures and adequate levels of autonomy are some of the examples of an enabling environment advanced by the WHO framework. The creation of such an environment has been reported as essential for building strong management of health systems [7, 11]. While this is widely documented, less attention has been directed towards effective approaches for achieving an enabling environment [11].

In sum, the WHO framework and existing literature proposes four main prerequisites for strengthening management capacity [7]. Similarly, several approaches have been advanced to develop these conditions [25]. However, each of these approaches is insufficient in and of themselves to result in well-managed health systems, and instead must be adopted in concert [22]. Thus, whereas the literature provides an in-depth analyses of the prerequisite conditions, approaches for meeting them and a basis upon which capacity building interventions can be evaluated against performance, it falls short of providing a synergistic framework upon which the development of competent managers can be based [11, 26]. Finally, literature on building health management capacity in low-income countries, especially at district level, is scarce [18]. In this study, we examined how district managers in a rural setting in Uganda perceived existing approaches to strengthening management so as to provide a pragmatic and synergistic model for improving management capacity building. Grounded Theory method allowed us to both investigate this question as well as construct a conceptual model based on the empirical material.

### District level management of health services in Uganda

Uganda is governed under a decentralized system through which the health services are delivered to the population. Administratively, Uganda is divided into districts that are further sub divided into counties, sub-counties, parishes and villages. Under the decentralized system, districts have increased in number from 45 in 1997 to 112 districts in 2014. This increase has been linked to the failures in the decentralization system [27]. Consequently, a recent desire for recentralization in Uganda has been noted, however in this paper we shall restrict ourselves to describing the National health system with a focus on the district level [28].

The highest level of referral exists at national level with specialized centers such as the National Cancer Institute and the Uganda Heart Institute to support the national referral hospital. This is followed by regional referral hospitals. At the district and below there exist general hospitals, Health Center IVs (HCIVs), Health Center IIIs, (HCIIIs) Health Center IIs (HCIIs) and Health Center Is (HCIs).

The Constitution (1995) and the local government act (1997) mandate the local governments (LGs) to plan, budget and implement health policies and health sector plans. The LGs implement these through the health department, which is headed by a district health officer. The health department is comprised of several players heading different health programs of the districts; these form the district health teams (DHTs). The DHT is supported by the district health management team (DHMT), which includes the district health (DHT), health center managers, and select members from other departments, political and administrative leaders, representatives from the private health providers and local NGOs.

The district health officer (DHO) heads the DHMT and is directly responsible for planning, implementation and monitoring health service delivery in the district. The DHO usually has a medical degree and a Master’s degree in Public Health (MPH) to be able to qualify for the position. However because of the numerous districts created over the last decade, it is not uncommon to find persons without formal public health training at masters level acting in this position for extended (more than 3 years) periods of time in some rural districts. The DHT consists of about 15 persons charged with different technical programs in the DHO’s office. These are usually appointed on the basis of their technical background and experience and rarely on managerial experience or training.

**Fig. 1:**
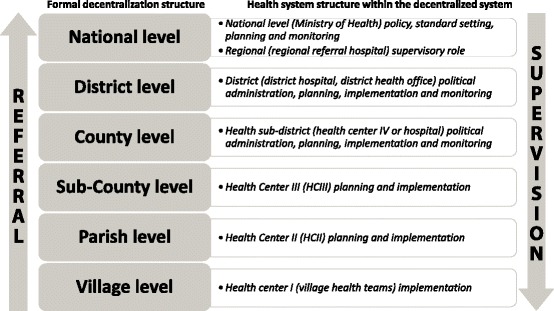
Levels of decentralization in relation to the national health system structure. Levels of decentralization in relation to the national health system structure in Uganda. The figure depicts the high level of management functions concentrated at the district level and at the levels below it in the health system under at decentralized system governance

At the health sub-district (HSD) level, a medical officer is responsible for managing service delivery and supervising other lower health centers. The HSD is either headed at a general hospital or at a HCIV. These medical officers are usually freshly qualified doctors with little or no management training and work experience, although in theory the policy dictates that they should hold an MPH. At sub county and parish levels, clinical staff usually with no managerial qualifications and with limited experience head the health centers. Important to note is that at HCIVs and below, the management role is simply an extra assignment and not a formal remunerated position within the human resource structure. Lastly, the community health worker or Village Health Team (VHT) heads the HCI, which is not, a physical structure but rather a team of volunteers charged with the responsibility of health mobilization and sensitization. This lack of managerial experience poses challenges to the system, as performance cannot be expected at optimum level. Therefore, several managerial issues exist in the health system at district level. Figure 1 is an illustration of the levels of decentralization in relation to the national health system structure [27, 28].

## Methods

### Description of study area

The study was conducted in three rural eastern Uganda districts – Kamuli, Pallisa and Kibuku. Uganda’s territory is 240,038 km^2^ of which 82% is covered by land. Whereas the national population is 37 million inhabitants, the district of Kamuli has 486,319, Pallisa has 386,890 and Kibuku has 202,033 inhabitants. All three districts have a fertility rate average of 6 children per woman [29]. These districts are characterized of slow growing townships with opportunities for small scale trading while the main livelihood is subsistence farming.

The health services in all three districts are administered in line with the decentralized health system described above. In addition, the three districts receive support from external partners, such as non-governmental organizations, international donor agencies, and academic institutions that focus on strengthening specific aspects of the health system or response to specific health conditions. These partners implement interventions in parts of the districts at different levels, usually through creating separate structures either within or without the formal health system structure. Makerere University School of Public Health (MakSPH), an academic institution to which authors, MT, EEK and SNK belong is one of such partners.

At the time of the study, MakSPH was implementing a 4 year project called MANIFEST aimed at increasing access to maternal and neonatal health services in the three districts using a participatory action research approach. The project team worked closely with the district stakeholders to stimulate demand for maternal health services at community level as well as to improve on the quality of health services offered. The uniqueness of the MANIFEST project lay in its approach, which allowed the local district level stakeholders to lead the implementation of the project activities while the external project team played a supportive role. According to the informants in this study, such an approach had previously not been used in the three districts.

### Study design

Kathy Charmaz's qualitative constructivist approach to grounded theory was used to conduct the study. This method was selected in order to capture local stakeholders’ own perceptions and definitions of management capacity building as well as approaches used to achieve this at the district level. The study began with some sensitizing concepts found in existing literature. While these concepts helped get the research started, the interviewing process remained open to exploring informants own definitions and understandings of the subject [30].

### Informants’ selection

Informants were purposively selected from three different types of district managers: district level administrative and political managers, district level health managers and health facility managers also known as facility in-chargers. District level administrative and political managers selected included the district political heads, the chief administrative officers, and select district council members. District-level health mangers comprised the district health officers and specific program managers such as the district health educators, the district senior nursing officers and the district health information and management systems officers. Then lastly, health facility managers at HCIII and HCIV level were interviewed.

All informants were chosen because they held key managerial positions at district level and in the health facilities that provide direct services to the population. For the facility managers, those who had been in service for at least 2 years were selected, as they were assumed to have a richer understanding and experience of the management function at their level. The facility level managers selected for this study came from both the intervention and comparison areas of the MANIFEST study. Table 1 is a summary of the informants’ characteristics.

**Table 1:** Summary of informants’ characteristics

Type of informants	Sex	Management training at time of appointment/ assignment	Age of informants Mean (Range)	Number of years of experience in management Mean (Range)	Professional discipline of managers
District level administrative and political managers *n* = 5	F: 1	Yes: 3	46.2 (37–54)	7.4 (5–.10)	
	M: 4	No: 2			
District level health managers *n* = 6	F: 2	Yes: 3	45.2 (35–60)	7.5 (3–15)	Medical officers =3
	M: 4	No: 3			Nursing officers = 3
Health facility managers *n* = 11	F: 5	Yes: 0	37.6 (27–54)	5.5 (2.5–11)	Medical officers = 1
	M: 6	No: 11			Clinical officers = 8
					Nursing officers = 2
Total *n* = 22	F: 8	Yes: 6	41.6 (27–60)	6.5 (2.5–15)	
	M: 14	No: 16			

Typical health managers in this study were middle-aged, mostly males and had been in the role for over 6 years at the time of data collection. Managers generally did not have formal management training at the time of being assigned or appointed into the management role

### Data collection

Data were collected using intensive interviewing, based on a guide with semi-structured questions [30]. The authors developed a specific guide based on the research question. On average, the interviews took approximately 45 min each. The interviewer [MT] adopted a conversational approach guided by sensitizing concepts (understanding of management, management capacity building, efforts undertaken to improve one’s management capacity, pros and cons of each of the efforts and preference for the efforts) in the interview guide as well as the direction of the responses from the informants. Each interview began by asking the informants to describe what their work usually involved as managers, and from that point on, the interviewer elicited more details while paying attention to new themes as well as ensuring that every informant covered all sensitizing concepts relevant to the subject of inquiry.

Initially, two informants across the three districts were selected from each type of managers. These initial interviews informed the direction of the sampling, which is referred to as theoretical sampling in the grounded theory methodology [30]. In order to capture richer perceptions on management capacity building at district level; a decision was taken to interview additional health facility managers rather than the administrative and political managers. As the later were found to posses limited knowledge and experiences of the subject matter although they played key roles in the entire process as shall be seen in the results and decision sections.

In total, 22 informants were interviewed: eleven facility managers, six district managers and five district level administrative and political managers. During data collection, memos were written on emerging codes that helped inform the direction of the interviews as well as the selection of the next informants. Saturation was reached by the 19^th^ interview in the MANIFEST intervention areas, which meant that emerging categories were full [30]. Three more interviews were conducted in areas where the MANIFEST intervention was not being implemented in order to see whether these differed theoretically from the previous interviews. They did not and indeed they supported the emerging model. At this point a decision was made to stop collecting any more data. The same principle of saturation was applied during data analysis.

### Data analysis

The data analysis process was a continuous process of reflection and comparison between empirical findings and emerging codes, beginning from the time of data collection [30]. Interviews were transcribed by research assistants and safely stored in a computer folder and backed up. To check for accuracy, the first author listened to all the audio recordings while reading the transcripts before they were stored. The transcripts were read and re-read entirely to obtain an overall picture of the interviews and to get familiar with the data.

The constant comparative method of analysis was continued with the transcribed interviews using MAXQDA a qualitative analysis software version 11.2. The next stage of analysis started by doing open coding (without any pre-determined codes) line by line and paragraph by paragraph in some instances [31]. Open codes were developed from and kept closest to the raw data with a very low level of abstraction. MT performed the coding, working closely with the last author. They shared and discussed the codes with the other authors so as to allow the examining of the codes in relation to the interview transcripts. This also ensured that the emerging analysis was grounded in the empirical data. The open codes were then grouped into clusters that related to each other and labeled. During focused coding, these labeled clusters were then used to re-examine the transcripts while concentrating the analysis on the selected concepts and sharpening them.

During theoretical coding, four concepts were established: a competent health manager, professionalizing health managers, engaging learning approaches and a supportive work environment. The linkages between these theoretical codes were examined along with their relation to the theoretical codes families proposed by Glaser [31]. The theoretical code family of “processes” best fitted the findings as it clarified health managers’ perceptions of what mattered regarding health management and building management capacity. A model that captured the iterative process of building a competent health manager was reconstructed.

Finally, the findings were compared with the WHO framework [7] and existing literature on management of health systems, as presented in the discussion section. Table 2 attempts to depict the movement from open codes to focused codes and to the theoretical codes.

**Table 2:** An Illustration of the coding process

Open codes	Focused codes	Theoretical codes
Defining management, reporting, supporting human resources, offering service to communities, being informed, planning, coordinating resources, conflict resolution, budgeting and resource control, controlling, collaborating with others, representing others, having knowledge, being in control, managing others, using data, listening to local news, reading research reports, reviewing data, being knowledge.	• Understands his or her roles well. • Is well informed. • Is empowered to execute management functions.	*A competent health manager*
Feeling appreciated, feeling unappreciated, competing interests of managers, having skills gaps, challenges faced by managers, benefits of training, limitations of training, having no choice, feeling overburdened, and having a conflict of interest.	• Formalization of management. • Conscious career choice. • Formal training.	*Professionalizing health managers*
Feedback sharing, support supervision, mentoring, holding meetings, importance of meetings, challenges of holding meetings, noting the benefits of workshops, noting the limitations of workshops, ensuring continuity, preference for multiple and engaging approaches to learning, learning through experience, learning from others, learning by practicing, and learning by doing.	•Mentoring and supportive supervision. • Quality regular meetings. • Specific in-service trainings. • Learning by doing. • Continuous learning atmosphere.	*Engaging learning approaches*
Strengthening teamwork, involving other stakeholders, having collective responsibility, learning from others, Receiving external support, enabling conditions, disabling conditions, improving working conditions, being supported, being monitored, being accountable, having conflicts, negative influences.	• Teamwork. • External support and oversight. • Empowering condition.	*Supportive work environment*

Table depicts the movement from open codes to focused codes and to the theoretical codes in the analysis process. The theoretical codes where achieved through a back and forth process between the focused codes, the open codes and the original transcripts in order to keep the integrity of the model

### Methodological considerations

The trustworthiness of our analysis and the resulting model can be assessed according to four criteria [30]. Our analysis and resulting model have credibility because these were achieved through an iterative process of getting familiar with the setting of the study and the data collected. Specifically, theoretical sampling, a back and forth process of analysis and data collection supported by memo writing was used. The principle of theoretical saturation was adhered to during the data collection to ensure that the theoretical categories in the model were fully explored. MT, EEK and SNK had been working with many of the respondents of the study for over five years as part of MakSPH’s support to districts. This presented an opportunity for MT to build good rapport with the informants and bring a solid understanding of the local context within which the health managers worked.

Our analysis and resulting model has resonance because these were achieved through an iterative process that yielded theoretical saturation at both data collection and analysis. The differing and rich experiences of the managers were sufficiently reflected in the model as depicted in the results section of this paper. To further strengthen the resonance of the model, a preliminary model was shared with the study participants for reflection and accreditation. Further research is recommended to allow an actual reflection on the competencies that these different sub-processes that make up the model can actually build among health managers.

Our analysis and resulting model have originality because they bring together several approaches that have previously been used and presented in either isolation or in combination. The model therefore advances interventions designed to build health managers’ capacity, which will invariably contribute to the strengthening of weak health systems in low-income countries. The model in addition provides distinctive prospects for different stakeholders to contribute to the process of building a competent health manager.

Finally, our analysis and resulting model is modifiable. It may be used in, and modified by studies in other settings and among other cases. This model proposes the combined application of previously disjointed strategies for building health management competencies and enriches the existing literature of strengthening health systems. The authors of the model therefore acknowledge the possibilities of being fine-tuned through its practical application elsewhere. Importantly, the model needs to be tested to get a higher understanding of its applications in the real world.

## Results

**Fig. 2:**
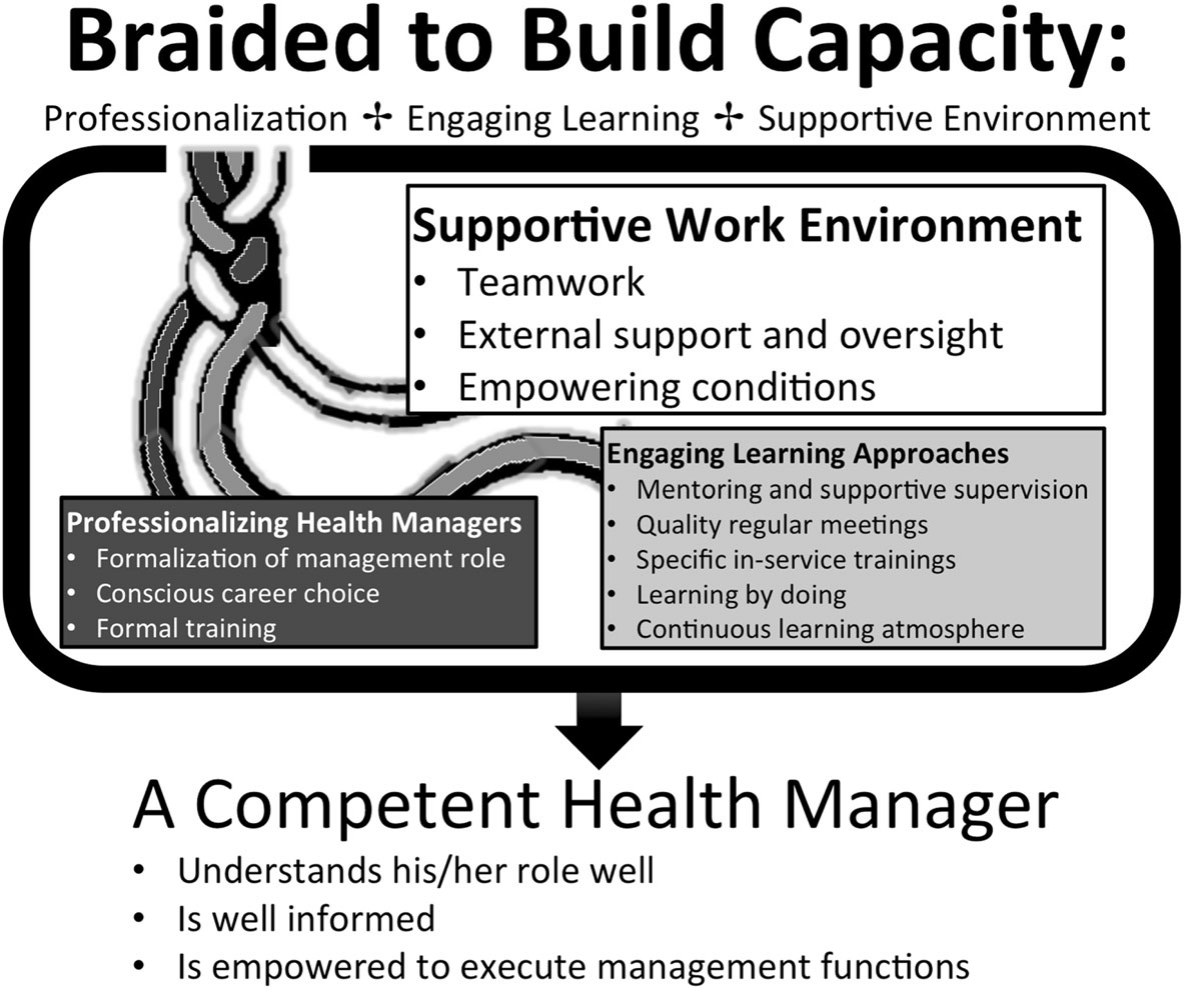
Building a competent health manager. An illustration of the interconnected reconstructed model of building a competent health manager at district level. The different sizes of the rectangles depict different amounts of time and effort investments needed to attain the different sub-processes of building a competent health manager. In addition, the model is iterative, dynamic and complex as depicted by the braided strands of the model

Through analyzing the interviews and constructing theory, we developed a model that explained health management capacity building as a social process integrating three sub-processes. The overarching process, which we named “building a competent health manager”, was made up of three sub-processes: a) professionalizing health managers, b) engaging learning approaches, and c) supportive work environment. The sub-processes varied from one another in that they required different amounts of investments (time, effort) to be accomplished. We portray this with different sizes of rectangles in the model, the bigger the rectangle, the more time and effort investment required to attain the sub-process (Fig. 2). Nonetheless, the sub-processes were connected to one another in that they reinforced one another in an integrated, and iterative manner, depicted by the braided strands in the model (Fig. 2). Therefore, no single sub-process was enough on its own, but rather all three were noted to be needed and ideally working in synergy to ensure the process of building a competent health manager. Figure 2 is an illustration of the model of building a competent health manager.

### A competent health manager

This category comprised three properties that defined what was viewed as a competent health manager. The category is the end result of the three sub-processes of building a competent health manager. The properties of a competent health manager included: having a good understanding of the meaning of management and its roles, being well informed as a manager and being empowered to execute management functions.

A good understanding of management roles was noted to be critical for a competent manager. This was shown by a wide range of roles that managers found themselves in, often without anticipating them as shall be seen in the sub-process of professionalizing health managers. Roles ranged from simple ones, such as work distribution, delegation, use of resources and directing, to more demanding and complex ones, such as drawing and executing work plans within available resources, lobbying for more resources, collaborating with other stakeholders, promoting team work, conflict resolution, and ensuring quality service provision. The quote below illustrated the roles of health managers.

*“The manager should be able to efficiently and effectively implement or carry out daily business or carry out an activity in abide to achieve better quality services using resources like people and money” (A District level administrative and political manager).*

A competent manager was also viewed as one who was well informed in his or her area of service delivery. Being well informed was regarded as providing a platform upon which managers could make decisions as well as guide others. To keep themselves well informed, managers rely upon the health system data, research studies and local news. Nonetheless, the use of health system data and research by health managers to make decisions was noted as rare at district level. The perception of managers on being well informed was illustrated in the following quote.

*“I have come to learn that as a manager I have to be knowledgeable, having facts. Data is very important in management whereby if you collect data and analyze it, it can help you identify what the problem is, so that you can get a solution to it. You can not lead others when you don’t have knowledge, it’s very difficult” (A Health facility manager).*

Lastly, being empowered meant being able to execute management functions as well as having the authority and resources to undertake one’s roles. The informants described management functions as consisting of coordination, planning, controlling, budgeting, reporting, conflict resolution and representation. According to the informants, a competent manager was one who was empowered to undertake these management functions.

*“I have to ensure that the facility is running normally, that there is good relationship between the community and my staff and the security of my staff. I budget for the resources and make sure that we spend according to our plan. Then we also report to the sub county authorities, the district and even the ministry of health. If I can not perform these duties, then I will have failed as a manager” (A health facility manager).*

### Professionalizing health managers

This category comprised of three properties. It entailed the formal recognition of health manager’s role within the health system at all relevant levels, making an active career choice to become a health manager and it further involved professional development of this choice by obtaining formal academic preparation. Compared to the other two sub-processes below, we interpreted this category as a sub-process that formed the foundation for building a competent health manager and required the least investment (time, effort) to achieve.

The need to professionalize health managers was drawn from a backdrop of health managers’ experiences. Most of them felt that the management function currently received relatively less attention and recognition. The recognition of the role of health managers at all levels of the health system was deemed essential for professionalizing health managers. The informants lauded this as a means of legitimizing and clarifying health managers’ roles as well as motivating them. This recognition was also viewed as enabling the process of making an informed career decision especially for the health facility health managers.

Having the freedom to consciously make a career choice to become a manager highlighted the fact that some health managers felt compelled or forced into the role. Informants explained that at the time of being assigned a management role, most of them were simply clinicians straight from school with very limited exposure to management, with some expressing disinterest in the role. This posed challenges to their identity as well as their productivity as managers who had officially been appointed as clinicians rather than managers. Feelings of frustration, failure and being overwhelmed were experienced especially among the health facility managers. For some managers especially at facility level, they would rather be clinicians seeing patients. Nonetheless, other managers clearly would wish to develop their careers in the direction of management. However, this needs to be by choice as well as by official appointment as noted by the informants.

*“Like I said, it’s just an extra assignment which was given to me without my choice. Really I don’t see the incentive, even if I do more than what the other clinicians do. Because if it were clinical work, it would be very easy to perform and you run away, you just see your patients and you go away which is why I was appointed” (A health facility manager).*

Finally, given the limited experience that most managers had when they were first appointed or assigned the role, obtaining academic preparation was viewed as critical to building health managers capacity at district level. Formal training was seen as offering informants an opportunity to appreciate the concept and roles of management. It serves as an essential introduction to the functions and tools of management, which the informants thought should then be supplemented by other engaging learning approaches. Nonetheless, some health managers also regarded formal training as a pathway for career progression and not necessarily a learning tool.

*“So when it comes to the academic training in management, it give’s one a bigger understanding of what management really means, most of us know nothing about management, so it is good to be exposed to that kind of training. And then the rest can be added on, like mentoring, workshops or even supervision. But of course some people just want papers for promotion not really to learn to do things better”(A district level health manager).*

### Engaging learning approaches

This category showed opportunities for practice and skill development and consisted of five properties: mentoring and supportive supervision, regular quality staff meetings, specific in-service trainings, learning by doing and a continuous learning atmosphere. We interpreted this category as a sub-process that forms the inside contents of building health managers capacity, and relative to the previous sub-process of professionalization, requires more investment in terms of time and effort, thereby increasing in complexity.

The use of mentoring and supportive supervision offers opportunities for feedback sharing and application of skills or knowledge. The next quotation demonstrated the positive aspects of mentoring and supervision as articulated by the informants:

*“Mentoring challenges me to improve on my knowledge in different things. You find that the DHO can send you to a meeting to represent him and you have to show that you know what you are talking about. So once the DHO guides me on what to do, I usually find no problem and he supervises my work as well, which is good because it makes one accountable” (A district level health manager).*

However, managers who were more concerned with career progression found mentoring and supportive supervision deficient in providing a clear career progress path. Similarly, informants disliked the fault-finding kind of support supervision.

Having quality meetings on a regular basis was another approach within the sub process of engaging learning approaches. The quality of meetings was largely defined as those promoting free and open discussions among participants as well as yielding productive action points. Meetings were seen as opportunities for addressing staff issues, reflecting on progress and sharing information. Nonetheless, if not well managed, meetings were viewed as tending to favor the out spoken over the reserved staff members. The informants expressed their belief in meetings helping to build management capacity in the quote below:

*“Actually meetings also are helping me to review our progress; it helps you understand how much you have achieved. And sometimes you find obstacles hindering you from achieving certain things and you redesign the way you plan and do things through sharing with others in meetings” (A health facility manager).*

Similarly, in-service trainings were viewed as aiding to build specific management skills and fix specific competency problems. The managers for example noted that workshops that were organized to help them with accounting and procurement challenges were useful in improving their management competencies. Workshops were also seen as refreshing and motivational as they take managers away from their usual places of work for short periods of time, such as 1 to 3 days. Nonetheless some limitations of workshops were noted: some participants have low levels of concentration in workshops and only view workshops as avenues for extra earnings given the relatively low salaries paid to health workers. The role of workshops in building competent health managers was elucidated in the following citation:

*“Workshops can refresh your mind, you can be on duty and you feel it is too much for you. Because these places are too remote, no electricity, my family is far from here, no TV. It feels like you are in a dark corner. So the workshops really motivate us, like I learnt how to account for money spent in my first workshop, we also get some little allowance when we go. So it helps to meet some costs, as you know our salaries are very small. By the time you come back, you feel somehow, you are motivated” (A health facility manager).*

Additionally, learning by doing was another engaging learning approach that was viewed as characterized by being given the opportunity to learn on the job. This required being adequately engaged in the process of undertaking certain activities to ensure that managers learned how to perform them as they actually undertake the activities. Informants noted that being engaged is a motivation in itself because it creates opportunities to learn from one’s mistakes and be more reflective as a manager. The quote below revealed the usefulness of learning by doing opportunities:

*“It is practical, it is very, very practical. Someone will come down, tell you lets us write a report, lets fill this register together. Like we recently received a hands-on training from our partners on procurement skills for our health managers. You see if you teach me while am doing something, it very difficult for me to forget” (A District level administrative and political manager).*

This sub-process was noted to be completed by a continuous learning atmosphere. The use of engaging learning approaches implied that learning is a continuous and iterative process, thereby promoting an attitude of patience and tolerance among health managers with respect to building their capacity. Building the competence of others was also perceived as a way of ensuring continuity of management roles or functions. This is related to the next sub-process of having a supportive work environment. Managers depicted the need for an atmosphere that supports unceasing learning in the citation below:

*“With management capacity we have to continue with the mentorship, we have to continue with learning new skills, supervision, the implementation and monitoring. Then we have to involve others so that there is continuity, learning cannot stop, when am away it doesn’t mean that things must stop” (A District level health manager).*

Lastly, this sub-process embraced a synergistic and iterative use of engaging learning approaches. Combined multiple approaches were considered needed not only to learn different competencies but also to reinforce lessons learned from any one of the approaches. No single approach was perceived to be sufficient to build all the needed competencies adequately; rather a combination created a greater synergistic effect.

### Having a supportive work environment

This category consisted of three properties: teamwork, external support and oversight and empowering conditions. We interpreted this sub-process as that which formed the frame for building health managers capacity and required the greatest amount of time and effort investment, therefore was the most complex.

Promoting teamwork was believed to lend greater legitimacy to managers and gives them more authority over the team. It was also seen as a means of sharing responsibilities, learning from others and having collective responsibility over the functioning of a health facility or district. Promoting teamwork entailed that managers were open to other team members’ ideas, empowered them by delegating duties and offered support to others. Similarly, it involved outward looking strategies such as collaborating with others, being transparent, appreciating others and sharing challenges with others to enhance the spirit of teamwork.

*“As a manager, I simply don’t do things alone, but other people do things on my behalf as well, which makes life a lot easier. So we work together and give each other feedback, which helps us to improve. For example when we needed to repair the door to the laboratory, I called them and said look we need to repair our door but the plan and the funds we have allow only for the motorcycle repair, can we reallocate?” (A health facility manager).*

Receiving external support and oversight was another aspect identified in the sub-process of having a supportive environment. By external, the informants meant anything that was outside their sphere of direct influence. While receiving external support seemed to suggest a docile health manager on the receiving end, informants noted that it was the explicit role of the manager to lobby and harness resources both from within and without the district. Receiving external support and oversight had two meanings. One meaning was receiving external projects or implementing partners that supported capacity building programs or health interventions in general. The other was having external oversight from a higher level of authority in the health system, which was viewed as creating a sense of responsibility, offers monitoring oversight and motivates the managers.

For example, working with local communities ease’s the pressure on the management to undertake community mobilization and awareness-raising activities. Meanwhile, political leaders can use their leadership authority and appeal to support these community-based activities, which in a way expands resources available to managers. The quotes below were an illustration of the provision of external support:

*“I have a very good chairman of the health unit management committee, he is very straight, for him he will tell you off, A is A and B is B, this is right, this is wrong. So he is very helpful when we have meetings and others are not supporting the right things. He also helps to resolve conflicts within the community especially when it involves the health workers” (A health facility manager).*

*“You know for us politicians wherever we are, whether in church or burial places, we are given the opportunity to speak, so during our speeches, we also sensitize people on health issues. So I feel like really having various stakeholders on board helps the health managers to do their work” (A District level administrative and Political manager).*

While external support was viewed as useful in supplementing government efforts and enabling the managers to meet their targets, the managers were quick to note it’s down side. For example at the end of externally funded projects, a reverse effect of benefits gained was noted to occur when communities and local systems find it difficult to continue on their own.

Having empowering conditions was the final aspect of the supportive work environment for health managers. This referred to situations that managers found useful in enabling them to successfully undertake their roles and become more competent managers. These conditions comprised: the promotion of local solutions, having resources, upholding local interests, being able to allocate/reallocate resources and learning in a familiar environment. These conditions were noted as partly enhanced by the engaging learning approaches described above but also by organizational structures. In contrast, disempowering conditions such as working in an unpleasant environment, facing negative political influence and frustrating local systems were viewed as working against the development of a competent health manager. Health managers as well as other stakeholders were viewed as playing important roles in creating these empowering conditions. For example it was perceived that, a supportive work environment required that managers were able to scan their environment and adapt to existing conditions and that local leaders provided support to resolve conflicts, sensitize communities and make managers feel welcome in a community by applying relevant approaches and organizational policies respectively. The next quotes illustrated the importance of empowering conditions in building competent health managers.

*“You know sometimes if you are on the ground, you come to know what works out and what does not work out and somehow you learn how to make things work according to the local conditions. You can even see how to increase your own resources, so the working environment is important, those politicians, the councilors and even the community they have to support you otherwise things are bad” (A health facility manger).*

*“When I was transferred, I went to facility A, the councilors there said, I was a very difficult person, after sometime I was transferred to facility B, in B, they were saying that we are not working. I was transferred again and then to another facility and now I am here and people are still complaining. I have had a very rough time being an in-charge. I need to rest from this work” (A health facility manager).*

## Discussion

Through the study, we developed a model that depicts building health managers’ capacity, as a complex process comprised of three interconnected, iterative, dynamic and complex sub-processes. Building a competent health manager according to the model requires a synergy of many different approaches and players with different roles. This does not happen in a linear or additive manner but rather in an iterative process that involves making pragmatic choices.

In the final stage of grounded theory method, we compare our model to the WHO framework presented in the introduction as well as existing literature on the topic. The World Health Organization framework consists of four key conditions needed to attain strong management for health systems: having, an adequate number of trained managers, managers with appropriate competencies, management support systems in place and an enabling work environment [7]. Our model enhances the WHO framework by drawing upon district health managers’ own perceptions and definitions to re-construct how these four conditions could be attained through three sub-processes.

The first sub-process of professionalizing health managers captures the importance of having adequate numbers of trained managers, competent health managers and management support systems. Whereas earlier studies emphasize mainly academic training as the means for professionalization [10, 23], our study found that this was important but not sufficient to ensure professionalization. Health professionals in this study were also motivated to become district-level health mangers when the health system recognized their role and enabled them to consciously choose or not the management role [17]. One of the key frustrations for health facility managers in this study was their “dual” role of management and offering clinical services simultaneously while their official appointments only reflected the latter.

This sub-process captures how to ensure the right persons for the management role, potentially generate higher job commitment, improve time and resource allocation to management roles, and in turn, improve performance of health managers [18]. Consequently, this sub-process indicates how to increase adequate preparation for the management role and its support systems by improving the professionalization of the role or career path and thereby creating demand for the role. While the discussion about professionalizing health management has been around for over three decades now, it’s not devoid of conflicting views [32, 33]. The proponents view it as a means of finding the best people to undertake this critical aspect of health care provision, while the opponents think of it as creating conflict between health care professionals and managers and view management as an ambiguous discipline [32, 33]. Despite these on going debates, we found in this study that dedicated effort to ensuring that health managers feel recognized, are motivated to undertake managerial roles and have basic management training relevant to health care was viewed as essential for better performance of health systems.

The second sub-process of using engaging learning approaches captures the importance of creating various opportunities for the development of management competencies. The sub-process is not identified in the WHO framework, though it is clearly implicit. As stated above, existing literature shows that strategies to build management competencies rely mainly on the use of academic training [10]. Engaging learning approaches have most frequently been used in community-based interventions and rarely for the development of management competencies within the health sector [34]. These approaches have been praised for stimulating learning through interactions across different stakeholders, offering opportunities for reflection, stimulating action-oriented change and empowering participants [35, 36]. Our findings suggest that the use of engaging learning approaches is not only appropriate for the development of management competences but also, through their synergistic application, has the potential to harnesses the various benefits of different approaches as found by other studies [37, 38].

The last sub-process of having a supportive work environment captures the importance of an enabling environment. Just as our study found, the WHO framework points to regular meetings, support supervision and mentoring, incentives, teamwork and adequate levels of autonomy as some of the examples of an enabling environment [7]. Nonetheless, some strategies for creating an enabling environment have also been shown to create unfavorable work environments if wrongly applied. For example, as our study shows, health managers did not find faultfinding and controlling supervision to be empowering [39]. In addition, they viewed engaging learning approaches as not relevant for climbing the career ladder. A reflection on experience and competencies in using engaging learning approaches for career progression is worthwhile as this study found them essential in building management competencies. Lastly, while the benefits of an enabling environment have been widely documented, less attention has been directed towards which approaches create an enabling environment for health managers [7, 11]. Our model shows that the use of engaging learning approaches provide opportunities to enhance supportive work environments for health managers along side structural policies.

The model developed in our study makes four central contributions to enhance the WHO framework and the existing literature. First, it emphasizes management capacity building as an iterative, dynamic and complex process rather than a set of characteristics of competent managers. Second, our model captures the need for professionalization of health managers at different levels of the health system. Third, our model indicates the benefits of engaging learning approaches through prolonged and sustained processes that act in synergy with one another. Fourth, our model shows that different amounts of time and effort are needed to create a competent health manager through an iterative, dynamic and complex process.

### Study limitations

The high level of abstraction in grounded theory could have led to missing out of specific contextual details that could have been captured by other qualitative methods. Nonetheless the abstraction gained here to develop the model can now be tested in specific contexts. In addition, a preliminary model was shared with the respondents to ensure the grounding of the model in the empirical data. Secondly, in this study, the sampling was done purposively, only informants that could provide the needed data to explore management capacity building for health managers in the three rural districts were selected. For example only facility health managers with at least 2 years experience were selected. Further insights from newly recruited managers could have been missed but the selected managers shared some of their earlier experiences to make up for this.

## Conclusions

The model of building a competent health manager at district level could be applicable not only in Uganda but also in other low-income settings. Reflecting on capacity building as a process rather than a set of competencies or characteristics of well performing managers was found to be fundamental in this study. A critical reflection on the roles of different stakeholders in this process is important for actualizing the implementation of the model proposed in this study. Training institutions, development partners, local and central governments, health workers, funding agencies and implementing partners are some of the key stakeholders in this process.

To professionalize health managers at district level, governments through parliaments and line ministries need to consider policy issues surrounding training of health managers, formalizing them in the health system structure at all relevant levels and creating a conducive structural environment for them. Similarly, training institutions are challenged with providing the appropriate curriculum for the different levels of management as well as supporting the target population to make informed career choices. This should be matched with adequate resource allocation to the process of professionalization that should be considered in critical review of arguments for and against it. This study found that recognizing the management role and providing support for the adequate preparation for this role is essential for district level managers.

In the same vein, key stakeholders should harness the strength created by deliberate use of supervision, mentoring, meetings, learning by doing and a continuous learning atmosphere to build management capacity. Higher-level health managers, development partners and governments need to consider sufficient allocation of resources to these approaches. According to the model developed in this study, engaging learning approaches need more time and effort allocation relative to professionalization of health managers given their iterative, dynamic and complex nature.

Finally, creating a supportive environment for the health managers at district level should be a mandate of all stakeholders concerned. These range from higher-level managers and development partners, to local opinion leaders, politicians and community members. Conditions surrounding district level managers should enable them to exercise adequate authority over resources allocated to them as well as challenge them enough to utilize the resources optimally while creating more. As noted in this study, the need to deliberately create a positive working environment for the health managers cannot be over emphasized; this should be matched with the greatest amount of time and effort allocation to the sub-process.
